# Does the anteromedial plate position affect proximal screw length and worsen the clinical outcomes in medial opening wedge high tibial osteotomy?

**DOI:** 10.1186/s12891-022-06080-4

**Published:** 2023-01-07

**Authors:** Dong Jin Ryu, Sang Jun Park, Dae Hee Lee, Kyeu-Back Kwon, Geun Hong Choi, Il Su Kim, Joon Ho Wang

**Affiliations:** 1 Department of Orthopaedic Surgery, Inha University Hospital, Inha University school of Medicine, Incheon, South Korea; 2 Department of Orthopaedic Surgery, Cheongju Micro Hospital, Cheongju-si, Chung-cheong bukdo South Korea; 3 Department of Orthopaedic Surgery, Samsung Medical Center, Sungkyunkwan University School of Medicine, Seoul, 06351 South Korea; 4 Department of Orthopaedic Surgery, Samsung Bone Hospital, Osan-si, Gyeonggi-do South Korea; 5 Department of Orthopaedic Surgery, Samsung Maditop hospital, Sungnam-si, Gyeonggi-do South Korea; 6 Department of Health Sciences and Technology, SAIHST, Sungkyunkwan University, Seoul, South Korea; 7 Department of Medical Device Management and Research, SAIHST, Sungkyunkwan University, Seoul, South Korea

**Keywords:** High tibial osteotomy, Plate position, Screw length, Lateral hinge fracture, Clinical outcome

## Abstract

**Background:**

During medial opening wedge high tibial osteotomy (MOWHTO), sometimes the plate tends to be positioned anteromedially. The plate position can affect the length of the proximal screw, which significantly affects stability after osteotomy. Therefore, research on the correlation among plate position, screw length, and clinical outcomes is needed.

**Methods:**

This retrospective review examines 196 knees in 175 patients who underwent MOWHTO from May 2012 to December 2018, for symptomatic medial compartment osteoarthritis with a varus alignment of > 5°. We evaluated the anteroposterior plate position, length of proximal screw, and postoperative computed tomography (CT). We reviewed patients’ clinical outcome scores, presence of lateral hinge fracture, neurovascular complications, and infection. The correlation among proximal plate position, proximal screw length, and clinical outcomes was evaluated using Pearson’s correlation analysis. A subgroup analysis by screw angle (> 48 ° or < 48 °) was also performed using chi-square test and Student t-test.

**Results:**

The mean proximal plate position was 16.28% (range, 5.17–44.74) of the proximal tibia’s anterior-to-posterior distance ratio, and the proximal screw length averaged 63.8 mm (range, 44–80 mm). Proximal posteromedial plate position and proximal screw length were significantly correlated (r2 = 0.667, *P* < .001), as were screw angle and length (r2 = 0.746, *P* < .001). Medial plating (< 48°) can use a longer proximal screw; nevertheless, no significant difference occurred in clinical outcomes between the two groups. Also, no differences occurred in complication rate, including hinge fracture.

**Conclusion:**

With more medially positioned plating during MOWHTO, we can use longer proximal screws. However, there was no significant difference in clinical outcomes and the incidence of lateral hinge fractures regardless of plate position and screw length.

## Background

High tibial osteotomy (HTO) is an effective surgical treatment option for mild-to-moderate medial compartment osteoarthritis (OA) of the knee and varus deformity [1, 2]. The objective of HTO is to realign the mechanical axis to delay the progression of arthritis [3, 4]. Currently, medial opening wedge HTO (MOWHTO) is performed more often than is lateral closing wedge HTO [5], because it offers benefits such as easier modification for alignment, a lesser likelihood of developing complications (e.g., peroneal nerve injury), and preservation of bone stock [6]. However, various issues have been reported, including failure to relieve arthritic pain, correction loss, plate or screw breakage, lateral hinge fracture, and nonunion [7–10].

Early rehabilitation after MOWHTO has been encouraged to prevent deep vein thrombosis, recover range of motion (ROM), and prevent disuse osteoporosis which is associated with the patient’s rapid return to daily life [11–14]. For early full-weight bearing without hinge fracture or screw breakage, the mechanical stability of the plate fixation must be achieved [15].

Although some biomechanical studies and finite-element analyses were reported [16], there were only a few clinical studies about the plate position, screw length, and clinical outcomes. These clinical studies have reported different results for stability and clinical outcome after MOWHTO depending on the location of the plate [17, 18]. Lee et al. reported no significant difference in clinical results despite the change in plate position using various plate designs [17]. Meanwhile, Nakamura et al. reported that the medial plating with sufficient proximal screw length could support the fibula tip and increase mechanical stability for decreasing over-time posterior slope change and complications [18].

However, there is no study on clinical outcomes according to the plate position using the same plate design and gap-filling methods. Therefore, the aim of this study was to evaluate the clinical outcomes correlated with plate position and screw length. If there is a strong relationship between the plate position and the length of the proximal screw, it can be used as a reference for determining the exact plate position during operation. We hypothesize that the medial positioned plate allows for longer proximal screws than the anteromedial plate, and it can lead to better clinical outcomes.

## Methods

### Study patients

**Fig. 1:**
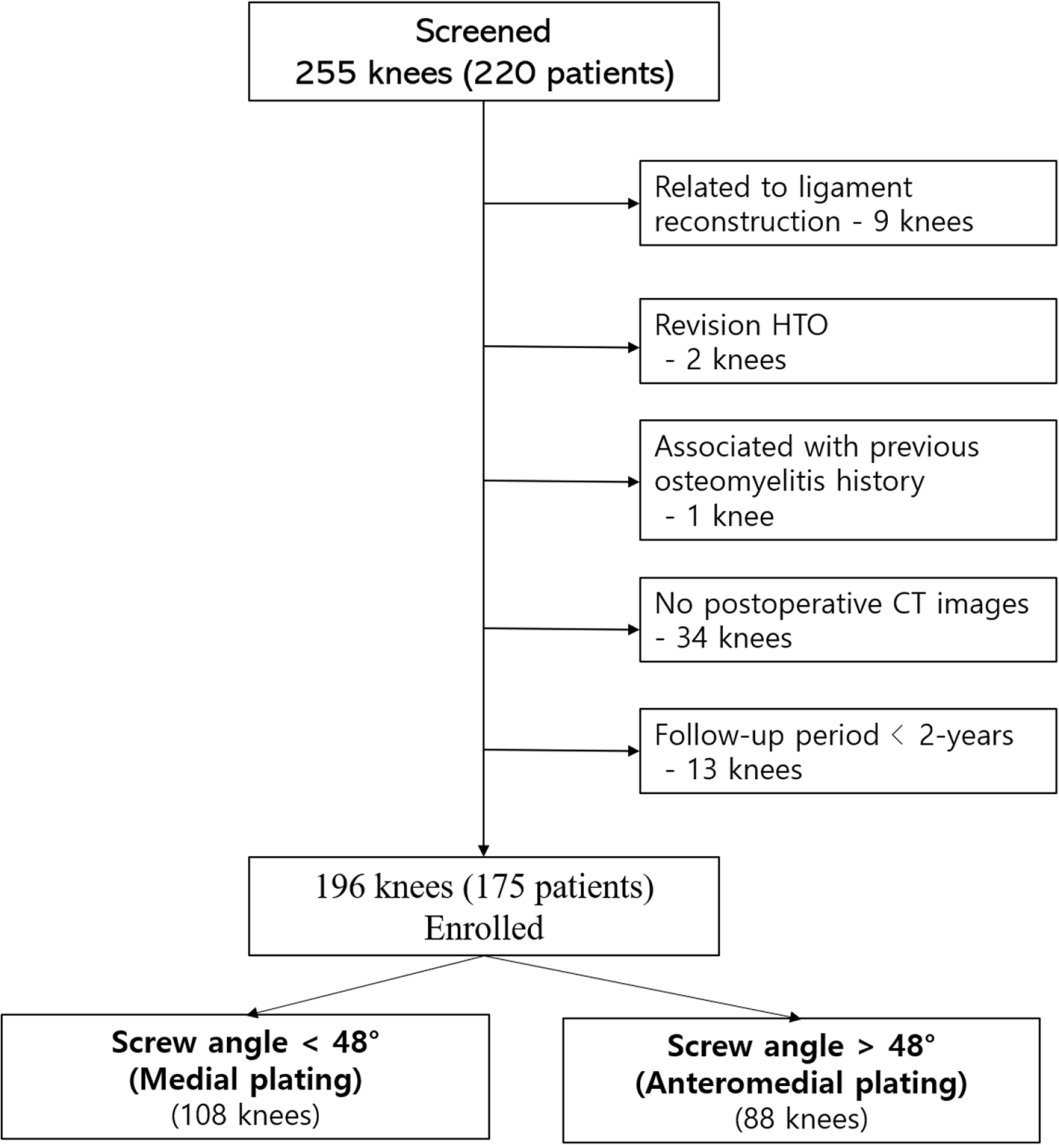
Flowchart of the study patients

This retrospective review included 266 knees in 231 patients who underwent MOWHTO from May 2012 to December 2018, for symptomatic medial compartment OA with varus alignment of > 5°. Inclusion criteria were as follows: (1) primary degenerative medial compartment OA (≦ Kellgren-Lawrence (K-L) grade 3) and (2) varus deformity of the lower extremity more than 5 degrees of hip-knee-ankle (HKA) angle. Exclusion criteria were as follows: (1) MOWHTO related to ligament reconstruction (9 knees), (2) revision MOWHTO (2 knees), (3) K-L grade IV with cartilage regeneration procedure (11 knees), (4) previous osteomyelitis history (1 knee), (5) No postoperative CT images (34 knees), (6) The follow-up period of < 2 years (13 knees) (Fig. 1).

This study protocol was approved by the Institutional Review Board of Samsung Medical Center (IRB No.2020–02–062-002). For this retrospective study, informed consent was exempted by investigational review board of Samsung Medical Center.

### Surgical techniques and postoperative rehabilitation protocols

The surgical technique followed the procedure previously described by Kyung and Ryu et al. [19, 20]. Two senior expert surgeons (JHW and DHL) performed all MOWHTO procedures. The targeted postoperative mechanical axis was 2–3° valgus [21]. The amount of coronal correction was determined preoperatively planning on the base of the standing whole leg, varus, and valgus stress radiograph [20, 22]. All osteotomies were performed in a biplanar fashion. The distal portion of the superficial medial collateral ligament (MCL) was transected along the planned osteotomy line at the tibia. After the mechanical axis was corrected by the navigation monitoring system (OrthoPilot Navigation System HTO version 2.1, Aesculap, Tuttlingen, Germany), and the proximal tibia was fixed using a TomoFix locking plate (Depuy Synthes, Bettlach, Switzerland) the medial osteotomy gap was filled with an allograft cancellous chip bone (Korea Bone Bank, Seoul, South Korea).

The patients were allowed to walk with crutches and toe-touch weight bearing on the operated limb immediately after the operation, and full ROM exercises began on postoperative Day 2. From postoperative Week 2, the patients were instructed to increase the amount of weight bearing on the operated limb gradually throughout the next 4 weeks [10, 23]. The patients visited the outpatient clinic at 6 weeks, 3 months, 6 months, and 1 year after the operation and annually thereafter. Radiography was taken at every visit, and standing whole leg radiographs were obtained after the patients could bear weight fully at 3 months postoperatively.

### Analysis of plate position and proximal screw length with CT

**Fig. 2:**
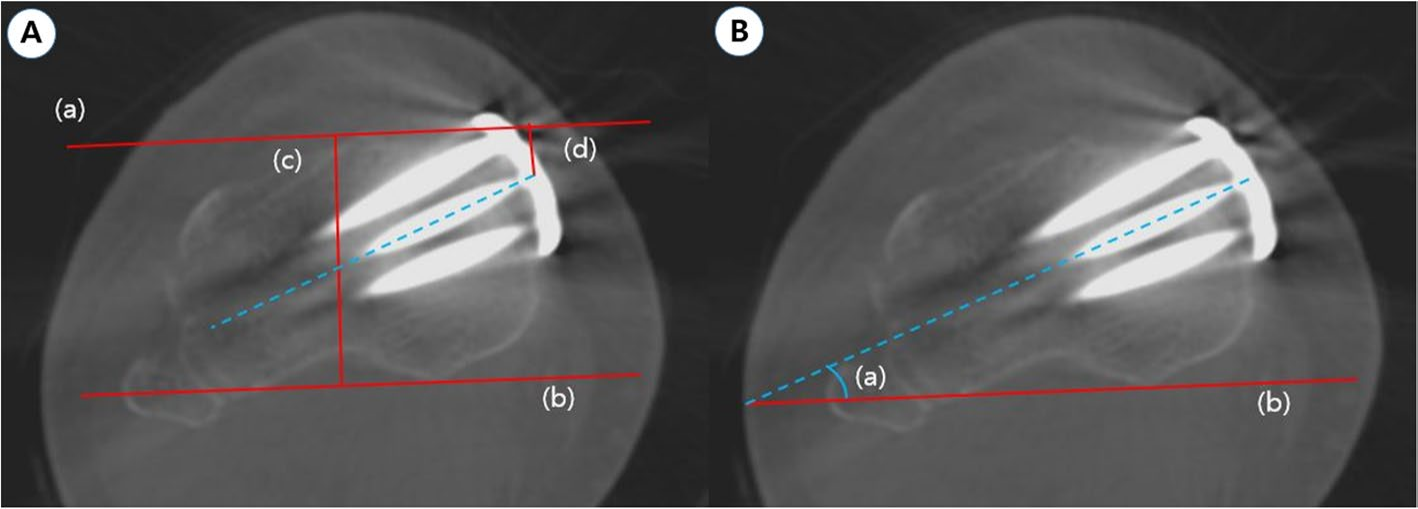
Measurement of plate position and screw angle in the proximal fragment. **a** A posterior line (*b*) crossing the posterior cortex of the proximal tibia was drawn and a second line (*a*) parallel to the posterior line and crossing the anterior surface of the proximal tibia was drawn. Anteroposterior (AP) position of the plate was evaluated by calculating the ratio between the distance of the anterior line to the plate center (*d*) and distance of AP proximal tibia length (*c*) was measured from the posterior line (b) to the anterior line (*a*). The plate position on the axial CT image was represented as a percentile from 0 (anterior) to 100 (posterior). **b** Measurement of the plate position in the screw angle. (*a*) The angle between the proximal central screw and the posterior cortex line (*b*)

Postoperative CT scans were achieved at 3 days after surgery. All CT data were acquired using the same CT scanner (LightSpeed VCT; GE Medical Systems, Milwaukee, WI, USA) slice thickness was 0.625 mm, and the collimation was 16 × 0.625 mm. Two trained orthopedic surgeons (DJR and SJP) evaluated the plate position in the proximal fragment and the screw angle in the axial CT views. We selected the axial cut where the three proximal screw heads and the plate junction were most visible. Proximal screw length was reviewed using the operative document. To estimate the proximal plate position and screw angle, we used the calculation method introduced by Lee et al. [17] (Fig. 2).

To measure the proximal plate position, the axial CT image was represented as a percentile from 0 (anterior) to 100 (posterior). (Fig. 2a) A posterior line (b) crossing the posterior cortex of the proximal tibia was drawn and a second line (a) parallel to the posterior line and crossing the anterior surface of the proximal tibia was drawn. We evaluated the anteroposterior position of the plate by calculating the ratio between the distance of the anterior line to the plate center(d), (d) and the distance of the anteroposterior proximal tibia length (c) was measured from the posterior line (b) to the anterior line (a). The screw angle was measured from the proximal tibia using the axial CT images (Fig. 2b). One line was drawn (b) through the posterior cortex, and a second line was drawn (a) crossing the center screw of the plate; the screw angle was formed by these two lines.

We divided the patients into two groups for subgroup analysis according to screw angle based on previous studies [17, 18]: the small-screw-angle group (< 48°, medial plating) and the large-screw-angle group (> 48°, anteromedial plating).

### Clinical outcomes

To examine the clinical outcomes, we evaluated the mean Lysholm score, International Knee Documentation Committee (IKDC) subjective score, and Tegner activity score preoperatively and at the final 2-year follow-up. We also reviewed the medical record and postoperative CT and X-rays during the follow-up period to investigate lateral hinge fracture, neurovascular injury, and infection.

### Statistical analysis

Two independent trained orthopedic surgeons (DJR and SJP) measured the radiographic parameters at intervals of three weeks between the measurements. We used the intraclass correlation coefficient (ICC) to determine intra- and inter-observer reliability, considering ICC values of > 0.75 to be good [24]. We used the average value of the measurements in the analysis. We adopted ICC > 0.8 for the reliability of the statistical analysis. All the parameters showed a good correlation.

To analyze the correlation among proximal plate position, proximal screw length, and clinical outcomes, we performed Pearson’s correlation analysis. In addition, we used a 2 × 2 chi-square test and Student t-test to evaluate the differences between the subgroups according to plate position (screw angle, 48°) [18]. We performed a post-hoc power analysis of subgroup analysis using G power 3.1. The examined statistical power was 0.97 (effect size: 1.124, α-error, 0.05). Statistical significance was considered at *P* < .05 at a confidence interval of 95%. We used SPSS version 25.0 (SPSS, Chicago, IL, USA) for the data analysis.

## Results

Detailed patient demographics are summarized in Table 1. The mean proximal plate position was 16.28% (range, 5.17–44.74) of the proximal tibia’s anterior-to-posterior distance ratio. The mean screw angle was 48.9° (range, 18–78°), and the proximal screw length was averaged at 63.8 mm (range, 44–80 mm). Proximal plate position and screw length were significantly correlated (r2 = 0.667, *P* < .001), as were screw angle and length (r2 = 0.746, *P* < .001).

**Table 1:** Patient Demographics

Patients (knees)	175 (196)
Age (years)	53.5 ± 8.1
Sex, male: female (n)	56: 119
Weight (kg)	70.8 ± 12.9
Height (cm)	161.3 ± 8.5
BMI (kg/m^2^)	27.1 ± 3.7

Values are shown as n or mean ± standard deviation. BMI, body mass index

Clinical outcomes improved in all patients compared to preoperative measurements. The mean Lysholm, IKDC subjective, and Tegner activity scores were 46.4 ± 22.8, 37.3 ± 18.2, and 2.6 ± 1.5, respectively, preoperatively and were 73.3 ± 21.0, 61.2 ± 19.0, 3.7 ± 1.4, respectively, at final follow-up (Table 2). The changes in clinical scores were significantly greater than the minimal clinically important difference (MCID) of each score (Lysholm: 10.1, IKDC subjective:11.1, Tegner activity score:1) [25]. In all patients, no conversion to arthroplasty was necessary, and no varus recurrence (HKA axis varus > 1°) or broken screws occurred at the 2-year follow-up.

**Table 2:** Pre- and Postoperative Clinical Outcomes

	Preoperative	Final Follow-up
Lysholm score	46.4 ± 22.8	73.3 ± 21.0
IKDC subjective score	37.3 ± 18.2	61.2 ± 19.0
Tegner activity score	2.6 ± 1.5	3.7 ± 1.4

Values are shown as mean ± standard deviation. All outcomes, *p* < .001. IKDC, International Knee Documentation Committee

We divided the patients into two groups for subgroup analysis on the basis of 48° of screw angle. We found no demographic differences between these groups. The proximal screw length was significantly longer in the medial plating group (< 48°); nevertheless, we found no significant differences in clinical outcomes between the groups (Table 3). In addition, no differences occurred in the complication rate, including hinge fracture.

**Table 3:** Subgroup Analysis Results According to Screw Angle

	Screw angle < 48° Medial plating (108 knees)	Screw angle ≥48° Anteromedial plating (88 knees)	*P* value
Age (years)	52.8 ± 7.7	54.7 ± 8.7	n.s.
Sex, male: female (n)	34: 74	27: 61	0.904
BMI (kg/m^2^)	27.3 ± 3.8	25.9 ± 3.4	n.s.
Proximal screw length (mm)	66.8 ± 6.2	59.0 ± 7.6	< 0.001
Lysholm score	72.9 ± 20.8	73.8 ± 21.7	0.116
IKDC subjective score	61.7 ± 19.1	60.9 ± 18.8	0.227
Tegner activity score	3.6 ± 1.4	3.9 ± 1.3	0.435
Complications			
Hinge fracture	15 (13.9%)	17 (19.3%)	0.39
Neurovascular injury	0	0	n.s
Infection	0	0	n.s

Values are shown as mean ± standard deviation except those for sex. IKDC, International Knee Documentation Committee

## Discussion

The most important finding of this study is that there were no differences in clinical outcomes, including clinical scores and lateral hinge fractures, regardless of plate position or proximal screw length. Therefore, our hypothesis was not validated.

Previously, several biomechanical studies have been conducted examining the effect of plate position. Takeuchi et al. [5] performed biomechanical tests using 28 sawbones tibia models with anteromedial and medial plate positions estimating the changes in tibial posterior slope angle and stress on the plate. These authors found that changes in both were significantly larger in an anteromedial plate group than in a medial plate group. Albornoz et al. [26] also conducted a biomechanical study using 15 sawbones tibia models with the anterior, anteromedial, and medial positions of the plate in the sagittal plane, demonstrating that stability increased as the plate was positioned in the medial. These studies suggest that medial plating is biomechanically superior, and a longer proximal screw length can maintain a more stable fixation [27].

However, clinical studies reported different results. Lee et al. have reported no significant clinical or radiological outcomes regardless of plate position and screw length [17]. However, this study used different plate contour designs. The plate with contours matched to the post- osteotomy condition was the most medial positioned and applied longer screw; however, there was no significant difference in clinical results. On the other hand, Nakamura et al. reported that medial plating using bone-substitute with deeper screw insertion reinforces the opening gap for better angular stability than anteromedial plating without bone-substitute [18]. However, as the bone substitute also contributes to stability [5], it is difficult to interpret it as a difference depending only on the plate position. In this study, the clinical results according to the plate position were evaluated under the same conditions, such as plate type and bone substitute. As a result, there was no significant difference. This result can be explained by the mechanical stability provided by the locking plate system, which is enough for the maintenance of the correction [17]. In previous biomechanical studies, the failure loads of the Tomofix plate were reported to be 2881 N, which is greater than the axial compressive force generated in the adult knee joint during level walking [28].

**Fig. 3:**
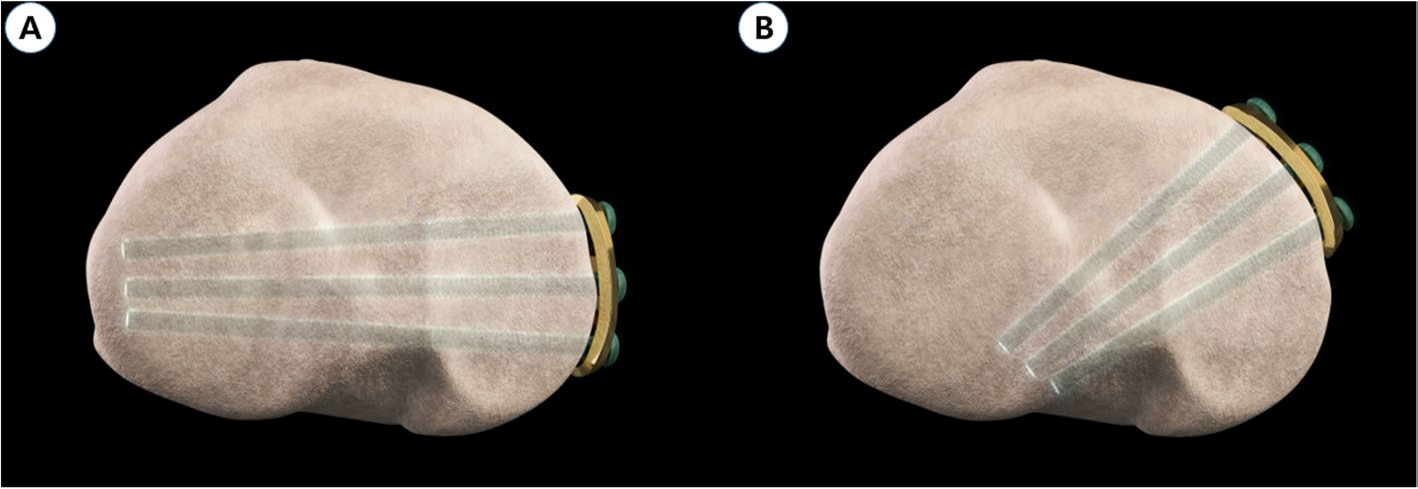
3D reconstruction images showing that (**a**) the plate is in the medial and the screw is long. in this position, the proximal screw can hold lateral lateral hinge area, (**b**) the plate is in the anterior and the screw is short

Inserting the proximal screw in the direction of the lateral hinge can reduce the stress on this lateral hinge [17, 29, 30]. In this study, we observed that medial plating significantly reduced the screw angles that indicate a screw direction toward the lateral hinge area. (Fig. 3) Thus, medial plating with the direction of the proximal screw facing the lateral hinge can offer mechanical stability after MOWHTO [17]. In addition, the screw direction toward the lateral hinge, not the posterolateral side can avoid neurovascular injury caused by proximal screw drilling [31]. Nevertheless, our study found no differences in complication rate according to plate position. With the comprehensive care during proximal screw drilling and the sufficient mechanical strength of the Tomofix plate may have resulted in the absence of observable differences in complications between the groups.

However, it is not easy to fix the plate by contouring it towards the medial side of the proximal tibia. Because of the inherent mismatch between the proximal and distal centers in the osteotomized tibia, it was difficult to position the conventional T-shaped plate medially [32]. Also, for the medial positioned plate, we should consider the more aggressive release of MCL [18]. There is still concern about the complete release of the superficial MCL, however, in a previously reported our study, the transection of the superficial MCL during MOWHTO does not increase valgus laxity [33].

Our study has several limitations. First, because of the retrospective design, the anatomical data of the patients who did not have a postoperative CT was unavailable; therefore, selection bias may have affected the results. Second, two senior surgeons performed the surgeries. Although both used the same surgical method, one of the senior authors used navigation, and the other performed the MOWHTO in a conventional way. Although there is not much effect on mechanical stability, there may be some effect on the clinical score [34]. Third, CT data could have a metal artifact; however, we used metal artifact reduction with optimizing threshold value. Fourth, we did not consider the anatomical variations of the tibia in the study patients, including shape, degree of tibia vara, and length of tibia plateau.

## Conclusions

With more medially positioned plating during MOWHTO, we can use longer proximal screws. The plate is likely positioned anteriorly when the proximal locking screw length is quite shorter than the pre-operative plan. In this case, we consider adjusting the plate position more medially. However, there was no significant difference in clinical outcomes and the incidence of lateral hinge fractures regardless of plate position and screw length.

## Data Availability

The datasets used and analysed during the current study are available from the corresponding authors upon reasonable request.

## Abbreviations

HTO: High tibial osteotomy
OA: Osteoarthritis
MOWHTO: Medial opening wedge high tibial osteotomy
ROM: Range of motion
K-L: Kellgren-Lawrence
HKA: Hip-knee-ankle
MCL: Medial collateral ligament
IKDC: International Knee Documentation Committee
ICC: Intraclass correlation coefficient
MCID: Minimal clinically important difference
